# Shifting brain inhibitory balance and connectivity of the prefrontal cortex of adults with autism spectrum disorder

**DOI:** 10.1038/tp.2017.104

**Published:** 2017-05-23

**Authors:** L A Ajram, J Horder, M A Mendez, A Galanopoulos, L P Brennan, R H Wichers, D M Robertson, C M Murphy, J Zinkstok, G Ivin, M Heasman, D Meek, M D Tricklebank, G J Barker, D J Lythgoe, R A E Edden, S C Williams, D G M Murphy, G M McAlonan

**Affiliations:** 1 Department of Forensic and Neurodevelopmental Sciences, The Sackler Centre for Translational Neurodevelopment, Institute of Psychiatry, Psychology and Neuroscience, King’s College London, London, UK; 2 Behavioural and Developmental Psychiatry Clinical Academic Group, South London and Maudsley NHS Trust, London, UK; 3 Pharmacy Department, South London and Maudsley NHS Foundation Trust, London, UK; 4 Department of Neuroimaging, Institute of Psychiatry, Psychology and Neuroscience, King’s College London, London, UK; 5 Department of Radiology and Radiological Science, Johns Hopkins University School of Medicine, Baltimore, MD, USA

## Abstract

Currently, there are no effective pharmacologic treatments for the core symptoms of autism spectrum disorder (ASD). There is, nevertheless, potential for progress. For example, recent evidence suggests that the excitatory (E) glutamate and inhibitory (I) GABA systems may be altered in ASD. However, no prior studies of ASD have examined the ‘responsivity’ of the E–I system to pharmacologic challenge; or whether E–I modulation alters abnormalities in functional connectivity of brain regions implicated in the disorder. Therefore, we used magnetic resonance spectroscopy ([1H]MRS) to measure prefrontal E–I flux in response to the glutamate and GABA acting drug riluzole in adult men with and without ASD. We compared the change in prefrontal ‘Inhibitory Index’—the GABA fraction within the pool of glutamate plus GABA metabolites—post riluzole challenge; and the impact of riluzole on differences in resting-state functional connectivity. Despite no baseline differences in E–I balance, there was a significant group difference in response to pharmacologic challenge. Riluzole increased the prefrontal cortex inhibitory index in ASD but decreased it in controls. There was also a significant group difference in prefrontal functional connectivity at baseline, which was abolished by riluzole within the ASD group. Our results also show, for we believe the first time in ASD, that E–I flux can be ‘shifted’ with a pharmacologic challenge, but that responsivity is significantly different from controls. Further, our initial evidence suggests that abnormalities in functional connectivity can be ‘normalised’ by targeting E–I, even in adults.

## Introduction

Autism spectrum disorder (ASD) is a neurodevelopmental condition, which impacts on three ‘core’ domains of function–social interaction, social communication and repetitive behaviours or interests. ASD has its origins in very early (prenatal) development, and persists through to adulthood.^1^ It is clinically diverse; the core difficulties can range in severity from mild to severe, and they are often accompanied by secondary conditions, such as anxiety and depression, especially in adulthood.^2^ This phenotypic variability likely reflects the marked aetiological heterogeneity of the disorder, and has made the identification of a common underlying pathology challenging. As a result, there are currently no effective pharmacological treatments for the core symptoms.

There is hope however, as genetic, post-mortem and preclinical laboratory studies indicate that the differences in the balance between the brain’s excitatory (E) glutamate and inhibitory (I) GABA systems may be one of the final ‘common pathways’ in ASD.^3^ This is perhaps especially true for GABA. For example, genetic studies have consistently implicated genes regulating GABA_A_ receptor expression with ASD,^4, 5^ whereas environmental risk factors for ASD, such as exposure to maternal inflammation in prenatal life, have been shown to disrupt gene expression across GABA pathways.^6, 7, 8^

Direct *in vivo* measurement of glutamate and, more recently, GABA, is now possible in humans using proton magnetic resonance spectroscopy ([1H]MRS). However, studies of glutamate and GABA at baseline in ASD are at variance.^9, 10, 11, 12, 13, 14^ For example, some have reported an excess of glutamate in the cortex^9^ and plasma,^15^ and in the amygdala and hippocampal regions.^10^ Conversely, we have reported lower levels of glutamate in the subcortex.^12^ GABA has been reported to be lower in the frontal cortex of children with ASD,^11^ yet higher in plasma.^16^ Still others have observed no differences in brain glutamate and GABA in ASD.^13, 17^

This divergence of findings may at least partly reflect between-study differences in (for example) the brain regions examined, age of participants, medication history and the presence of common comorbid mental health symptoms such as anxiety (mediated by GABA^18^). However, all prior studies examined E–I without challenging the system, and it is also possible that differences in glutamate and GABA ‘flux’ contribute to the variety of findings reported. This is because glutamate is a precursor of GABA, and there is continuous ‘flux’ between glutamate and glutamine, and glutamate and GABA in the brain.^19, 20, 21, 22, 23^ Glutamic acid decarboxylase (GAD) is a rate limiting enzyme in the glutamate/GABA-glutamate cycle, and has been reported to be abnormal in ASD.^24, 25^ Thus, E–I ‘dynamics’ (as opposed to the static state) may be critical in ASD—but this has never been directly studied *in vivo.*

Regional E–I dynamics may have widespread influence by regulating the functional connectivity of resting-state brain networks. For example, in typical individuals, prefrontal glutamate concentration has been shown to correlate with the strength of prefrontal functional connectivity;^26^ whereas local GABA concentration predicts the extent of inhibition within the motor network.^27^ However, although altered functional connectivity measures are commonly reported in ASD (for example, see refs 28, 29, 30), no-one has examined if these can be modulated through E–I.

Therefore, we conducted the present study to compare E–I and functional connectivity responsivity of the prefrontal cortex in adult men with and without ASD. We selected the prefrontal cortex as a region of interest (ROI), because this region is reliably implicated in the core symptoms in ASD, including deficits in theory of mind^31, 32^ and the regulation of socio-emotional responses.^33^ We used [1H]MRS and resting-state functional magnetic resonance imaging (fMRI) to measure (respectively) change in the E–I flux and functional connectivity of the prefrontal lobe following a pharmacological challenge with the drug riluzole. Our MRS measure of E–I flux was the fraction of GABA within the total measured pool of GABA plus glutamate and glutamine (Glx), which we defined as the ‘Inhibitory Index’. Our measure of functional connectivity was the strength of correlation (parameter estimate; PE) between activity in the prefrontal cortex and the rest of the brain.

We selected the drug riluzole because it has a broad range of actions at both GABA and glutamate targets,^34, 35^ and it should therefore modulate glutamate–GABA flux in a majority of individuals. For example, riluzole blocks the pre-synaptic release of glutamate, facilitates GABA_A_ receptor activity^36, 37^ and has been reported to shift glutamate–glutamine flux in bipolar disorder.^38^ Importantly, however, the pharmacokinetic and safety profile of riluzole is well-established; and a single oral dose of 50 mg riluzole, as used here, reaches a peak plasma concentration 1 h after oral administration^39^ and has a very low risk of adverse effects.

## Materials and methods

### Participants

*A priori* calculations indicated that a sample size of *n*=12 is sufficient to capture a 10% change in MRS measured metabolite concentration at 80% power, *α*=0.05;^40^ and this is in line with the minimum number needed for typical fMRI activations.^41^ However, to increase confidence in our results, a total of 37 adult males participated in the study; 17 individuals with ASD and 20 unaffected controls (Table 1). Each individual had two scans and so each acted as their own ‘control’ this reduced the inter-subject variability to improve power.

Individuals with ASD were recruited through the National Autism and ADHD Service for Adults at the Maudsley Hospital, London; a specialist diagnostic service for adults. All ASD individuals had a consensus clinical diagnosis of ASD led by a consultant psychiatrist using ICD10 research criteria;^42^ and reached Autism Diagnostic Observation Schedule (ADOS)^43^ algorithm cut-offs in both communication and social domains.

Inclusion criteria were: IQ score above 70; absence of current psychoactive medication; and being medication free for a minimum of 6 weeks, to avoid possible confounds from drug interactions. Exclusion criteria were: a comorbid psychiatric or medical disorder that might affect brain development, for example, epilepsy or psychosis, head injury, or any genetic or chromosomal disorder associated with ASD, for example, tuberous sclerosis or fragile X syndrome. The ASD group scored significantly higher (as expected) in their self-rated autism symptom scores (autism quotient; *P*<0.001), and anxiety (STAI; *P*<0.005), but the groups did not differ in age or IQ (*P*=0.44 and 0.15, respectively), see Table 1.

All participants provided written informed consent. Ethical approval for this study was provided by Camden and Islington London NHS Research Ethics Committee, study reference: 13/LO/0091.

### Procedure

Each participant was scanned on two occasions, one week apart. MRI scanning began 1 h after oral administration of a capsule containing either 50 mg riluzole, or an equivalent placebo. The order of administration was randomised and double-blind, so that neither the researcher nor participant knew which capsule was given. We further controlled for order effects by ensuring that approximately half the individuals in each group had placebo first and the other half had riluzole first.

### [1H]MRS data acquisition

[1H]MRS data were acquired on a 3 Tesla (3T) GE Excite II Magnetic Resonance Imaging scanner (GE Medical Systems, Milwaukee, WI, USA). The scanning protocol included an initial structural MRI scan, namely a 3D inversion recovery prepared fast spoiled gradient-recalled echo (IR-FSPGR) acquisition (number of slices=124, slice thickness=1.1 mm, inversion time (TI)=450 ms, repetition time (TR)=7.084 ms, echo time (TE)=2.84 ms, field of view=280 mm, flip angle=20°). This was then used to set the voxel location for the spectroscopy scans.

**Figure 1:**
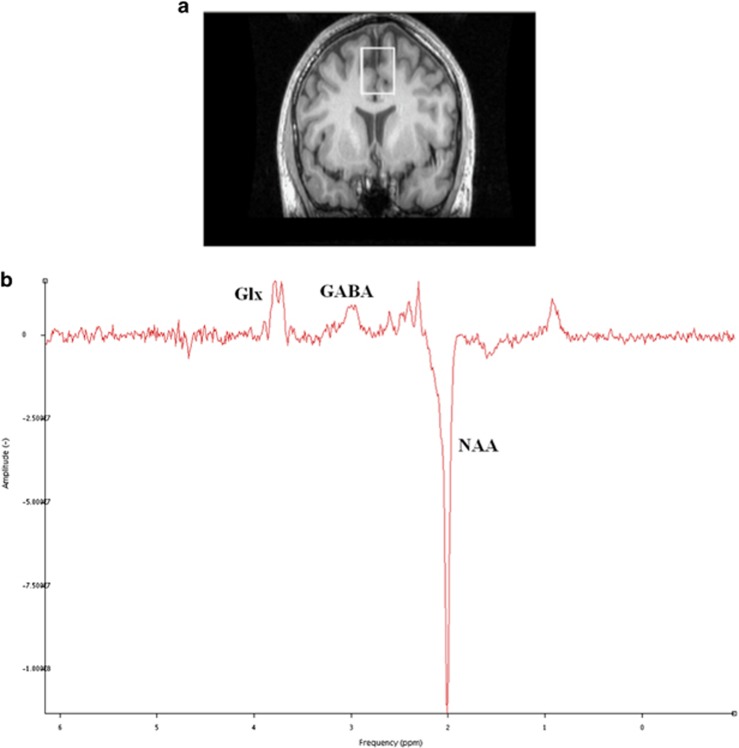
[1H]MRS voxel positions and example spectra. (**a**) Cortical region of interest; medial prefrontal cortex (mPFC) (25 × 30 × 40 mm^3^) outlined in white, comprising primarily of anterior cingulate cortex. (**b**) Example spectroscopy output from the prefrontal voxel (Glx (glutamate+glutamine); GABA; *N*-acetylaspartate (NAA) identified using jMRUI spectroscopy software.

Single voxel J-edited MEGAPRESS [1H]MRS spectra were then acquired in a prefrontal cortex region of interest using previously described methods.^12^ More specifically, we obtained spectra from the bilateral dorsomedial prefrontal cortex (25 × 30 × 40 mm^3^; TR=2000 ms, TE=68 ms), which included the anterior cingulate cortex. See Figure 1a for an illustration of the voxel placement.

### [1H]MRS data analysis

[1H]MRS spectra were pre-processed using GE SAGE software (SAGE 2007, General Electric Healthcare Technologies, Milwaukee, WI, USA) to perform an edit ON–edit OFF subtraction, to extract the unedited spectrum (equivalent to conventional PRESS spectrum) and also to extract the unsuppressed water spectrum. These spectra were then quantified using jMRUI version 4 software.^44^ Each [1H]MRS spectrum was manually checked for quality to ensure adequate signal-to-noise ratio and absence of artefacts (Figure 1b). If required, artefacts were removed using jMRUI peak fitting and removal algorithm, if this was sufficient to restore data integrity. Metabolite concentrations were estimated using the AMARES algorithm. In the subtraction spectrum; GABA (including macromolecule^45^) at 3 p.p.m., Glx (Glutamate+Glutamine; as the measure of glutamate) as two peaks at 3.8 and 3.75 p.p.m. and *N*-acetylaspartate (NAA) at 2.01 p.p.m. were estimated. In the unedited spectrum; choline at 3.19 p.p.m., creatine at 2.99 p.p.m. and NAA at 2.01 p.p.m. were estimated. Absolute metabolite concentrations (in institutional units) were then calculated by dividing the amplitude of each peak by the amplitude of the unsuppressed water peak from the water spectrum at 0.00 p.p.m.

To quantify E–I flux, an ‘Inhibitory Index’ was defined as the fraction of GABA relative to total GABA plus Glx, within each voxel, for each subject: that is, Inhibitory Index=GABA/(GABA+Glx). This inhibitory index does not represent an absolute ratio of GABA to glutamate molecules in the brain tissue, as [1H]MRS has different sensitivities for these two molecules. Rather, the inhibitory index is a relative measurement that theoretically ranges from 0 to 1. Therefore, a value approaching zero would indicate relatively more Glx (excitation); values close to 1 would indicate more GABA (inhibition).

### [1H]MRS voxel composition calculation

Group differences in proportion of white matter, grey matter and cerebrospinal fluid contained in the voxel (partial volume effects) are a potential confound for spectroscopy, and a particular consideration for the present study, as previous literature has found volumetric group differences between control and ASD subjects.^46^ The structural MRI was first segmented into grey matter, white matter and cerebrospinal fluid (CSF) using SPM2.^47^ The position of the [1H]MRS voxel was then registered to the corresponding segmented structural scan and the grey matter, white matter and CSF content of this region was calculated using in-house software. We found no group differences in the percentage composition of grey or white matter in the ROI, nor did this change in the presence of riluzole (Supplementary Table 1). There was however, a significant difference in the percentage CSF composition in ASD. In light of this, we corrected each metabolite for individual CSF levels prior to analysis and controlled for both water and CSF levels, as previously described.^12^

### Resting state fMRI BOLD data acquisition

Immediately after [1H]MRS acquisition, in the same scanning session, resting state functional MRI data were acquired. Resting state fMRI acquisition parameters were as follows: an echo-planar imaging (EPI) sequence, TR=2 s, TE=30 ms, 256 volumes. Slice thickness=3 mm, number of slices=38, slice spacing=0.3 mm. Each slice comprised of 64 × 64 voxels with a field of view of 240 mm, for a final voxel size of 3.75 mm × 3.75 mm × 3.3 mm. Pulse and respiration parameters were also acquired during the fMRI scan, for the purposes of correcting for physiological artefacts in the fMRI dataset, using a photoplethysmogram pulse oximeter and a chest band, respectively.

### Resting state fMRI BOLD data analysis

fMRI data were processed using FSL 5.0.0. The following pre-processing steps were applied: motion correction using MCFLIRT; slice-timing correction using Fourier-space time-series phase-shifting; non-brain removal using Brain Extraction Tool (BET); spatial smoothing using a Gaussian kernel of full width at half maximum (FWHM) 5 mm; grand-mean intensity normalisation of the entire 4D dataset by a single multiplicative factor; and highpass temporal filtering (Gaussian-weighted least-squares straight line fitting, with sigma=50.0 s).

Manual inspection and removal of artefactual components was then carried out using hypothesis-free probabilistic independent component analysis as implemented in MELODIC (multivariate exploratory linear decomposition into independent components) Version 3.10, part of FSL.^48^ The decisions as to whether to exclude each component from a given fMRI scan were made by a researcher blind to group and to drug status.

### Statistical analysis

Statistical analysis of demographic, questionnaire, and [1H]MRS data were performed using IBM SPSS v.22 software (IBM SPSS Statistics for Macintosh, Version 22.0. Armonk, NY, USA). Figures were generated using GraphPad Prism version 7 for Windows (GraphPad Software, La Jolla, CA, USA). Age, IQ and self-reported questionnaire group comparisons were performed using independent samples *t*-tests. The numbers included in each MR analysis depended upon a stringent quality check of data available for each participant per analyses at each time point.

#### [1H]MRS

The [1H]MRS voxel composition in each group was compared using repeated measures two-way ANOVAs with ‘group’ as between-subjects factor and ‘drug’ as within-subjects factor (control *n*=20, ASD *n*=17). The inhibitory index could be calculated at two time points from 27 participants (that is, 54 data-points) and was therefore compared using repeated measures two-way analysis of variance with ‘group’ as between-subjects factor and ‘drug’ as within-subjects factor. Where there was a significant group × drug interaction, we also tested for a group difference in the change in inhibitory index elicited by drug. The State score measure of anxiety was included as a covariate in these analyses, as anxiety is closely linked to GABA,^49^ and participants with ASD scored significantly higher than controls on measures of anxiety (Table 1).

*Post-hoc* the change in inhibitory index was converted to a *z*-score contribution so that the contribution of a change in glutamate to any change in the inhibitory index following riluzole could be explored using Pearson’s correlation analyses; and a Fisher ‘*r* to *z*’ transformation was used to compare correlation co-efficients between groups. For consistency across analyses, the State score measure of anxiety was included as a covariate. Finally, we screened for any unpredicted effects of group or drug on other metabolites measured (choline, creatine or *N*-acetylaspartate); any difference identified would have been subject to a threshold of *P*<0.01 to accommodate multiple comparisons.

#### Resting state fMRI connectivity

**Figure 2:**
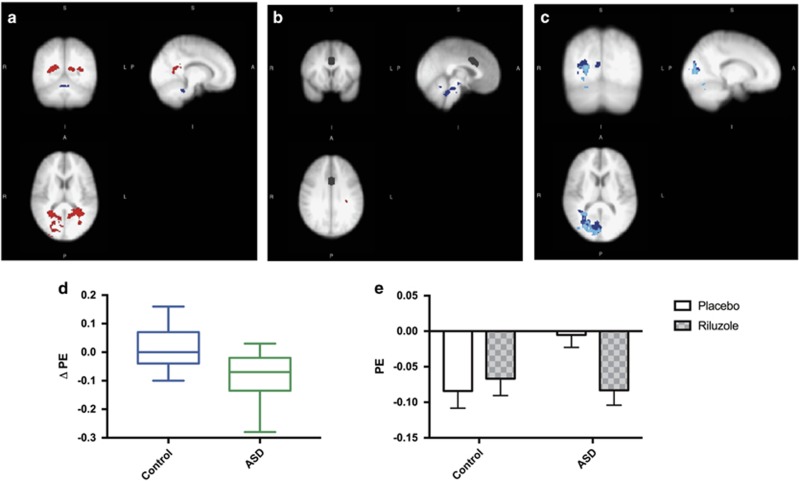
Functional connectivity differences between controls and ASD. (**a**) BASELINE: In the control group there was greater connectivity (an inverse correlation) between a prefrontal seed region and posterior regions compared to ASD (red). (**b**) The seed region (mPFC) is shown in black. (Also indicated is a restricted region of connectivity between mPFC and the brain stem/cerebellum (blue) in ASD at baseline). (**c**) RILUZOLE: Riluzole increased connectivity (an inverse correlation) between the prefrontal seed and posterior cortices in ASD only. Blue: riluzole<placebo (*P*<0.05, cluster-corrected). Light blue: voxels in which the within-subjects effect of riluzole vs. placebo is negative in ASD (*P*<0.05, cluster-corrected). (**d**) Change in functional connectivity elicited by Riluzole is significantly different between groups (*t*_(26)_=0.394, *P*<0.005). (**e**) Riluzole did not alter the functional connectivity of the prefrontal lobe in controls but ‘restored’ functional connectivity in the ASD group to control levels post riluzole (connectivity × group Interaction, two-way repeated measures ANOVA; F_(1, 26)_=9.696, *P*<0.005).

The anterior cingulate region contained within the dorsomedial prefrontal cortex voxel was used as the seed in a whole brain seed-voxel resting state fMRI connectivity analysis of *n*=18 controls and *n*=17 ASD subjects. The seed-voxel analysis was performed using a general linear model implemented in FEAT (FMRI expert analysis tool) version 5.98 (ref. 48) as follows: for each participant, the mean signal time course from an anterior cingulate seed region (contained within the prefrontal cortex MRS voxel; see Figure 2b was first extracted using the fslmeants tool. This time course was entered as an explanatory variable (EV) in FEAT, and the participant’s pulse, respiration, and the mean signal from grey, white, and CSF masks were entered as the five additional EVs of no interest. We then carried out higher-level fMRI analysis using FLAME (FMRIB’s local analysis of mixed effects) stage 1. Higher-level EVs included drug (riluzole or placebo) and group (ASD or control). The *Z* (Gaussianised T/F) statistic images were thresholded using clusters determined by *Z*>2.3 and a cluster-corrected significance threshold of *P*=0.05.^50^

PE values from the first level fMRI data, for each participant, were our measure of the mean connectivity between the seed and the network of voxels found at the group level (Figure 2a). High absolute PE values indicate high connectivity. Full details of the fMRI result can be found in Supplementary Table 2.

Potentially confounding differences in head movement were examined by estimating mean absolute displacement and mean relative volume-to-volume motion using FSL MCFLIRT. There were no group or drug effects on either parameter (all *P*>0.8).

All data are expressed as mean±s.e.m. unless otherwise specified. A significance threshold of *P*<0.05 was adopted for all pre-planned analyses.

## Results

### Men with ASD have significant differences in E–I responsivity

**Figure 3:**
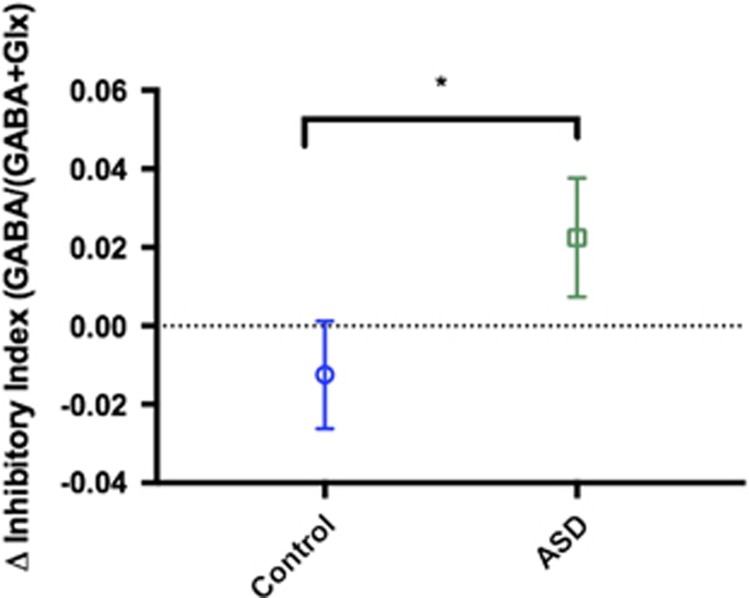
Riluzole increases inhibition in the prefrontal cortex of the autism spectrum disorder (ASD) group only. Riluzole increased the inhibitory index in the ASD group, not controls: *Drug × group interaction; F_(1, 24)_=4.288, *P*<0.05. *Post-hoc* analyses demonstrate a significant difference between groups in the change in inhibitory index after riluzole administration: ANCOVA; F_(1, 27)_=4.290, *P*<0.05. Results are expressed as mean (±s.e.m.) and are corrected for State anxiety score.

At baseline (that is, placebo), there were no group differences in the inhibitory index, or absolute levels of GABA or glutamate in the medial prefrontal cortex region. However, riluzole administration shifted the inhibitory index in opposite directions in the ASD and control groups. Specifically, riluzole increased the proportion of GABA in the prefrontal cortex of the ASD group but decreased the proportion of GABA in controls (group × drug interaction; F_(1, 24)_=4.288, *P*<0.05). This was confirmed by a significant group difference in the change in inhibitory index elicited by riluzole (F_(1, 27)_=4.29, *P*<0.05). There were no significant effects of group or drug on other metabolites measured (choline, creatine or *N*-acetylaspartate). Please see Figure 3.

### E–I balance is regulated differently in men with and without ASD

**Figure 4:**
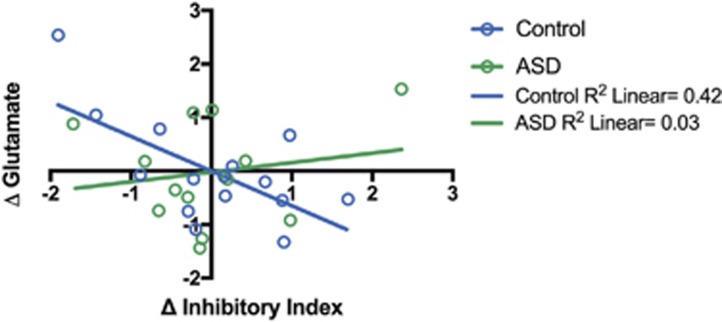
Inhibitory tone is regulated differently in men with and without autism spectrum disorder (ASD). The change in inhibitory index was negatively correlated with changes in Glx in controls (Pearson correlation; *r*=−0.705, *P*<0.01), but not men with ASD (*r*=0.357, *P*=0.28). Furthermore, there was a significant group difference in correlation coefficient (Fisher *r* to *z* transform, *z*=2.23, *P*<0.01). Results are expressed as *z*-scores ((value−mean)/standard deviation) and are corrected for State anxiety score.

In controls a decrease in inhibitory index (or GABA fraction) elicited by riluzole was accompanied by an increase in Glx (*r*=−0.705, *P*<0.01), see Figure 4. In ASD, the increase in the inhibitory index (or GABA fraction) was not linked to Glx. This relationship between the change in inhibitory index and change in glutamate measure in each group was significantly different (Fisher *r* to *z* transformation, *z*=2.23, *P*<0.01).

### Baseline functional connectivity differences in ASD are ‘normalised’ post riluzole

There was a significant group difference in the change in functional connectivity elicited by riluzole (*t*_(26)_=0.394, *P*<0.005). Specifically, riluzole increased the minimal/absent baseline prefrontal functional connectivity in the ASD group towards the control levels; and there was no longer a group difference in functional connectivity post riluzole (Figure 2).

## Discussion

To the best of our knowledge, this study provides the first direct evidence that responsivity of the E–I system and functional networks in ASD is regulated differently from controls. Specifically, in men with ASD the E–I acting drug riluzole increased a prefrontal cortex inhibitory index but decreased it in controls. Riluzole also increased prefrontal functional connectivity from ‘absent’ at baseline in ASD, to control levels.

We found no baseline differences in the level of cortical glutamate or GABA in men with ASD relative to controls. This is consistent with some (unpublished data and refs. 17, 51), but not all,^9^ previous studies. However, our results indicate that despite a comparable baseline, the E–I response to a riluzole challenge was diametrically opposite in men with ASD and controls. In controls, the decrease in the inhibitory index (or GABA fraction) was correlated with an increase in Glx. In ASD, riluzole increased the inhibitory index (or GABA fraction) without changing Glx. Overall, this pattern of results indicates that riluzole shifts flux towards GABA in ASD and towards glutamate–glutamine (Glx) in controls.

This unusual E–I responsivity in ASD may help explain other paradoxical findings from studies of the way people with ASD respond to E–I acting medications. For example, GABA_A_/benzodiazepine receptor agonists typically have an inhibitory effect in non-ASD populations, but can sometimes cause excitation in individuals with ASD.^52, 53^ This opposite direction of response in ASD is important because the initial selection of drug candidates for clinical trials in ASD is often based on their action in unaffected individuals. If, however, the nature of the brain response in ASD fundamentally differs from other populations then similar outcomes cannot be anticipated, and trials are likely to be unsuccessful. This exacerbates the challenges of identifying effective pharmacological treatments for ASD. For example, a number of clinical trials have been based on the presumption that drugs which increase inhibition in other populations should also be effective in ASD. These have included the anti-epileptic medication lamotrigine, which reduces glutamate release in the cortex, and d-cycloserine, a partial agonist at the NMDA glutamate receptor. However, neither approach has been successful in ASD.^54, 55, 56^

The cause of such E–I responsivity differences in ASD is unknown. However, they may have their origins in early developmental abnormalities, and perhaps especially in the GABA inhibitory system. For example, in ASD, it has been proposed that there is a delay or alteration in the typical ‘switch’ from an excitatory action of GABA in prenatal life, to its inhibitory action in the postnatal period.^57, 58, 59, 60^ There is also evidence from a genetic model of ASD, the neuroligin knock-in mouse, indicating that GABAergic, but not glutamatergic, signalling is enhanced in early postnatal development.^18^ Abnormalities in GABA pathways in ASD are also evident in later life, and these include a reduction in the levels of GAD type 65 and 67 in a number of brain regions,^24, 25^ and GABA receptor abnormalities in ASD, including in the anterior cingulate component of the prefrontal cortex voxel sampled here.^61, 62^ Thus, in ASD, early aberrations in E–I pathways may lead to persistent alterations, not necessarily in the absolute amount of GABA or glutamate but in their flux, receptor availability and action, and hence on their subsequent influence on brain network activity.

Consistent with this, our work suggests that the typical functional connectivity of a prefrontal-posterior brain network is absent in ASD. Similar baseline differences in resting state networks in ASD have been observed before, and our findings are consistent with the ‘long range underconnectivity’ reported in ASD (reviewed in Geschwind and Levitt^63^). Moreover, the dorsomedial prefrontal cortex/anterior cingulate region of interest examined in our study has been specifically identified as the critical node within a disconnected system which contributes to the social difficulties found in ASD.^63, 64^ This accumulation of observations of long-range underconnectivity in ASD has lead to the conceptualization of the condition as a ‘Developmental Disconnection Syndrome’.^63^

Developmental disconnection in ASD has been suggested to arise in two ways: (i) ‘a weakening of already formed connections’ or (ii) ‘a failure of certain connections to establish correct organisation *de novo*’.^63^ This then has implications for the severity of the ASD phenotype, with a failure to establish correct connections theroetically leading to more severe features. Here, riluzole (re-)established a functional relationship between the prefrontal and posterior cortices in high functioning adults with ASD which was similar to controls. This implies that any developmental disconnection in our cohort of autistic men is more likely to be ‘a weakening’ rather than an irreversible ‘failure’ of connectivity *per se*. In other words, the functional architecture of brain in ASD, though not measureable at baseline, was still in place and revealed by riluzole.

Clinically, riluzole has shown promise in reducing symptoms in some^65, 66^ but not all individuals with ASD.^67^ Thus, as expected in a spectrum condition like ASD, not everyone responds to the same extent to a drug challenge. In future studies, a neuroimaging approach such as the one used here could begin to help personalise medicine for ASD. For instance, future studies investigating clinical treatment with riluzole may be of interest in cohorts enriched with individuals who can be shown to mount a brain response to a single test dose.

The limitations of our study include the inherently limited spatial resolution of [1H]MRS, which restricts measurements to ‘bulk’ levels of metabolites. This macroscopic approach provides insight to the gross regional changes in E–I flux after challenge (see for example, Brennan *et al.*^38^), but does not allow for circuit level analysis. Future work is warranted to dissect the circuitry of the prefrontal cortex in more detail. One way of doing this may be to use more directed pharmacological probes, for example, specific to different GABA receptor sub-types.

We also acknowledge that, although based upon prior power calculations, the sample size for this study was still modest. This precluded further exploration of the relationship between symptom profile and E–I responsivity in ASD. Future work will be required to confirm the findings from this ‘Proof of Concept’ work, and determine if these are generalisable, for example, to females with ASD, to other age groups and/or to individuals with intellectual difficulties.

In conclusion, using [1H]MRS and fMRI, we found that E–I flux and functional connectivity of the prefrontal cortex are differentially regulated in adults with ASD compared with the controls. Importantly, inhibitory tone and functional connectivity can be shifted pharmacologically—and even in adults with ASD.

## Figures and Tables

**Table 1:** Participant demographic and clinical symptoms

	*ASD*	*Control*	T *(df)*	P*-value*
Number	17	20	N/A	
Age (years)	33 (2.5)	30 (1.9)	−0.79 (35)	0.44
FSIQ	114 (3.0)	120 (3.5)	1.48 (31)	0.15
AQ	32 (2.2)	13 (1.1)	−0.76 (24)	0
STAI, State	35 (3.3)	24 (0.8)	−3.3 (18)	0.004
OCI	25 (3.9)	7 (1.1)	−4.5 (19)	0
				
ADI-R (*n*=8) A domain	14 (3.4)			
ADI-R B domain	13 (2.2)	N/A		
ADI-R C domain	5 (0.8)			
				
ADOS (*n*=16) A domain	7 (0.7)	N/A		
ADOS B domain	4 (0.5)			

Abbreviations: ADI-R, Autism Diagnostic Interview—revised (Domain A, social interaction; Domain B, communication; Domain C, restrictive and repetitive patterns of behaviour); ADOS, Autism Diagnostic Observation Schedule; AQ, autism quotient; ASD, autism spectrum disorder; FSIQ, Full Scale Intelligence Quotient; N/A, not applicable; OCI, Obsessive Compulsive Inventory; STAI State, State/Trait Anxiety Inventory.

Groups differed in their neuropsychological tests of symptoms linked to ASD, but not in age or IQ score (Students *t*-test).
